# Acute effectiveness of a “fat-loss” product on substrate utilization, perception of hunger, mood state and rate of perceived exertion at rest and during exercise

**DOI:** 10.1186/s12970-015-0105-8

**Published:** 2015-11-25

**Authors:** Ahmad Alkhatib, Marcos Seijo, Eneko Larumbe, Fernando Naclerio

**Affiliations:** Division of Sport and Exercise Science, School of Social and Health Sciences, University of Abertay, Bell Street, Dundee City, DD1 1HG United Kingdom; Centre for Sport Science and Human Performance, School of Science, University of Greenwich, Medway Campus, Central Avenue, Chatham Maritime, Kent, ME4 4TB London, United Kingdom; Clinical Research Institute, Texas Tech University Health Sciences Center, Lubbock, TX USA

**Keywords:** Multi-ingredient, Weight loss, Thermogenic, Maximal fat oxidation

## Abstract

**Background:**

Achieving fat-loss outcomes by ingesting multi-ingredient mixtures may be further enhanced during exercise. This study tested the acute thermogenic effectiveness of a commercially available multi-ingredient product (Shred-Matrix®), containing Green Tea Extract, Yerba Maté, Guarana Seed Extract, Anhydrous caffeine, Saw palmetto, Fo-Ti, Eleuthero root, Cayenne Pepper, and Yohimbine HCI, on fatty acid oxidation (FAO), perception of hunger, mood state and rate of perceived exertion (RPE) at rest and during 30 min of submaximal exercise.

**Methods:**

Following institutional ethical approval, twelve healthy recreationally active participants, five females and seven males, were randomized to perform two separate experimental ergometry cycling trials, and to ingest 1.5 g (3 × capsules) of either a multi-ingredient supplement (SHRED) or placebo (PL). Participants rested for 3 h, before performing a 30-min cycling exercise corresponding to their individually-determined intensity based on their maximal fat oxidation (Fatmax). Fatty acid oxidation (FAO) was determined at rest, 3 h before exercise (Pre1), immediately before exercise (Pre2) and during exercise (Post), using expired gasses and indirect calorimetry. Rate of perceived exertion (RPE) was measured every 3 min during the 30-min exercise. Additionally both mood state and perception of hunger were assessed at Pre1, Pre2 and Post exercise. A repeated measures ANOVA design and Cohen’s d effect sizes were used to analyze potential differences between times and treatment conditions.

**Results:**

FAO increased in SHRED from Pre1 to Pre2 [0.56 ± 0.26 to 0.96 ± 0.37, (*p* = 0.003, d =1.34)] but not in PL [0.67 ± 0.25 to 0.74 ± 0.19, (*p* = 0.334) d = 0.49], with no differences were found between conditions (*p* = 0.12, d = 0.49). However, Cohen’s d = 0.77 revealed moderate effect size in favor of SHRED from Pre to Post exercise. RPE values were lower in SHRED compared to Pl (*p*< 0.001). Mood state and perception of hunger were not different between conditions, with no interaction effects. However, a trend was shown towards improved satiety in SHRED compared with PL, [F(1,11) = 3.58, *p* = 0.085].

**Conclusions:**

The multi-ingredient product’s potential enhancement of FAO during exercise, satiety, and RPE reduction suggests an acute effectiveness of SHRED in improving the exercise-related fat loss benefits.

## Background

Even though an improved diet and exercise are known to be the best tools to reduce excess body weight and reverse the associated health risks [1], the popularity of weight-loss aids have increased as a fast method to obtain the desired outcomes. Commercially available single or multi-ingredient supplements, especially herbal-based thermogenic products have been reported in healthy obese and normal weight individuals with a high degree of effectiveness on metabolism and weight loss [2, 3].

It is thought that the thermogenic effects of multi-ingredient mixtures such as those containing caffeine, guarana, ephedrine, capsaicin and green tea extracts act synergistically to increase fatty acid oxidation (FAO), energy expenditure (EE), resting metabolic rate (RMR), hemodynamics and sympathetic function following the ingestion [4–7]. For example, tea catechins and caffeine in green tea induce further thermogenic function beyond that is induced by caffeine alone; also the thermogenic effect of ephedrine can be potentiated by caffeine, primarily by enhancement of sympathetic neuronal release of norepinephrine and epinephrine [7]. Caffeine intake has been shown to be effective in enhancing the function of the central nervous system and stimulate fat metabolism and lipolysis based on its effects on the androgenic receptors and its actions as adenosine receptor antagonist that would speed up the activity of cells and prolong lipolysis [8].

Newly available commercial thermogenic supplements have introduced various blends in order to improve the caffeine-related effects on mood state, cognition, blood pressure and heart rate by inducing central-related psychomotor, and peripheral-elated physiological changes [8–10]. New products have also introduced new weight-loss properties such appetite control [11–13]. However, limited research on their effectiveness produced equivocal results. Hoffman et al. [10] and Outlaw et al. [11] tested two different commercially available products that were based on two different blends containing several thermogenic agents such as caffeine, ephedrine and green tea mixtures. Whilst both studies showed increased fat metabolism and energy expenditure at rest, Hoffman et al. reported increased tension and confusion [10]. Furthermore, subjective appetite has been shown to change following nutritional and supplementation interventions [12, 13]. However, it is still unknown whether and how appetite is affected by multi-ingredient weight loss supplements.

Recently validated visual analogue scales for measuring appetite may provide simplified and reliable results [14], which can be used to track the efficacy of ingested supplements on appetite.

The effects of exercise on metabolism are also well established. Metabolism is known to be stimulated several fold during exercise. It is also established that substrate utilization during exercise is mainly determined by exercise intensity [15]. The reliance on fatty acid oxidation (FAO) shifts towards carbohydrates (CHO) oxidation as the exercise intensity increases [15, 16]. The most established concept describing the effects of exercise on FAO and CHO is the Cross-Over concept [15], which defines the power output at the cross-over point (COP) where the EE from CHO fuels begin to predominate over that from fat fuels. Additionally, the exercise intensities at which the reliance on FAO reaches its maximum have been defined as maximal fat oxidation (Fatmax) [16–18]. Exercising at those intensities (i.e. Fatmax intensities) have been reported to be effective in enhancing a number of exercise and weight-loss outcomes, including enhanced glycogen sparing, delaying fatigue mechanisms, and weight and body fat reduction [19–21].

Despite its importance, the joint optimization of metabolism through combining the effects of exercise with those from weight-loss supplements containing effective active weight-loss ingredients is under-investigated. Previous work on multi-ingredient weight loss supplements have shown increases in EE over 2–3 h time periods following caffeine ingestion in combination with additional ingredients including ephedra, black tea, green tea extract, Citrus aurantium, and yohimbine [4–6, 10, 11, 22]. However, all of those studies were conducted in resting conditions, and only limited research has investigated the exercise-dependent metabolic effects. Alkhatib, [23] reported an augmented FAO and EE derived from FAO at low and moderate exercise intensities following the ingesting of 1 g of Yerba Maté after one hour of ingestion [23].

With newly available weight-loss multi-ingredient formulations that have not been previously investigated, it would be necessary to investigate specific exercise dependent metabolic outcomes in these supplements. More specifically investigating the potential metabolic effects with previously reported psychological outcomes and adding the measurements of appetite control. For example, Yerba Maté is reported to have an appetite suppression effects and promote satiety [24], and commonly believed to have a less jittery effects compared with caffeine [9].

Enhancing exercise dependent fat-loss, such as metabolic and psychomotor outcomes of

Fatmax intensities, satiety and mood state may be achieved by administering a combination of effective weight-loss ingredients such as those contained in a commercially available products, including Green Tea Extract, Yerba Maté, Guarana Seed Extract, Anhydrous caffeine, Saw palmetto, Fo-Ti, Eleuthero root, Cayenne Pepper, and Yohimbine HCI. Therefore, this study aims to determine whether and how a commercially-available product (Shred-Matrix®), containing the latter ingredients, affect the fat-loss outcomes during exercise including FAO, perception of hunger, mood state and rate of perceived exertion (RPE).

It is hypothesized that FAO during exercise, particularly at Fatmax intensities, perceived exertion, satiety and mood state profile are all enhanced following the ingestion of a multi-ingredient fat-loss product, compared with an inert placebo.

## Methods

The study followed a double-blind crossover repeated measures controlled design. Environmental conditions were maintained the same in all testing sessions (Mean ± SD: 20 ± 1 °C, 775.6 ± 12 mmHg and 51.1 ± 6.1 %) for air temperature, barometric pressure and relative humidity respectively. The University of Greenwich granted institutional ethical approval, and all experimental procedures were carried out in accordance with the ethical guidelines of the World Medical Association Declaration of Helsinki. All study participants provided their informed written consent based on university-approved documents after the purpose of the research and experimental procedures were explained.

The participants were twelve healthy recreationally active adults, five females and seven males [Mean ± SD: age = 24 ± 3.8 y, height = 174 ± 9.1 cm, body mass = 69 ± 17.0 kg, body mass index (BMI; in kg^.^m^−2^) = 22.5 ± 3.85, body fat percentage (Fat%) = 21.3 ± 9.6 %, systolic blood pressure (SBP) =1 20.1 ± 9.7 mmHg, diastolic blood pressure (DBP) = 71.8 ± 5.7 mmHg]. All participants were screened prior to the start of the testing in order to determine that they were free from illness and any type of orthopedic limitation or injury. Exclusion criteria were as follows: A) History of any cardiovascular or respiratory disease, hypertension, liver or kidney disease, musculoskeletal or neuromuscular or neurological disease, autoimmune disease, cancer, peptic ulcers or anemia. B) Taking medications (except contraceptive pills), including those for heart, pulmonary, thyroid, anti-hyperlipidemic, hypoglycemic, anti-hypertensive, endocrinologic, psychotropic, neuromuscular, neurological, or androgenic conditions, as well as a family history of heart problems, high blood pressure, and/or stroke, and being pregnant or breastfeeding. C) Consuming any ergogenic aid or above habitual caffeine consumption rate (200 mg.day^−1^) for at least 6 weeks prior to the study, based on all types of caffeinated beverages (coffee, energy drinks, soft drinks, caffeine supplements or medications). All Participants refrained from taking any supplements for the duration of the study and were instructed to refrain from strenuous exercise or alcohol and caffeine consumption for at least 24 h before each test. Participants have also completed a 3-day 24-hr food diary with details about serving amounts for breakfast, lunch, dinner, snacks and additional meals.

### Experimental procedures and protocols

All participants reported to the Physiology Laboratory on three separate occasions followed by 3 h fasting state in the first session, and 12 h overnight fast in the second and third sessions. Each testing session (between 8:30 and 11:30 am) was separated by at least three days within two weeks period. Session one was aimed to assess participant’s body composition and endurance performance relisted variables including peak oxygen uptake $\left(\overset{\cdotp }{\mathrm{V}},{\mathrm{O}}_{2\mathrm{peak}}\right)$ and the intensity associated with the maximum fat utilization (Fatmax). During the second and third visits, after assessment of body composition, participants were randomized to ingest 1.5 g (3 × capsules) of either a multi-ingredient supplement (SHRED), (Shred-Matrix®, Muscle-Pharm Corporation, USA), or a placebo (PL) containing maltodextrin and hemp protein powder, presented in similar-appearance capsules to SHRED. Three capsules with similar coatings of either SHRED or PL were placed within an empty water cup and taken in the same way with a 240 ml of water. The SHRED capsules contained Green Tea Extract, Yerba Maté, Guarana Seed Extract, Anhydrous caffeine, Saw palmetto, Fo-Ti, Eleuthero root, Cayenne Pepper, and Yohimbine HCI. The thermogenic ingredients per capsule included approximately 70 mg of green tea leaf, 50 mg caffeine anhydrase, and 100 mg of Guarana seed extract. The exact content and other ingredients are in a proprietary blend.

Immediately following the anthropometric and body composition assessments, and ingestion, participants rested for 150 min in a semi recumbent position in quiet laboratory condition. Resting measurements involved heart rate (HR), blood pressure BP [systolic (SBP) and diastolic (DBP)] and RMR. Additionally, mood state was assessed just after the ingestion (150 min before exercise), immediately pre and post exercise. Perception of hunger was assessed every 30 min after the ingestion, immediately pre and post exercise.

For the estimation of FAO and CHO at rest and during exercise, breath by breath cardiorespiratory measurements included oxygen uptake $\left(\overset{\cdotp }{\mathrm{V}},{\mathrm{O}}_2\right)$, carbon dioxide production $\left(\overset{\cdotp }{\mathrm{V}}\mathrm{C},{\mathrm{O}}_2\right)$ and respiratory exchange ratio (RER), using an online gas analyzer (Metalyzer Cortex 3B, Leipzig, Germany), which was fully calibrated as described in previous similar studies [23].

### Anthropometric and body composition assessments

Body mass (BM) and height were assessed, on a standard scale and stadiometer. Body composition was assessed for body fat percentage (%BF) by whole-body air displacement plethysmography (Bod Pod®, Life Measurements, Concord, CA), in accordance with the manufacturer’s instructions as detailed elsewhere [25]. Participants were tested wearing only tight fitting clothing (swimsuit or undergarments) and an acrylic swim cap. Thoracic gas volume was estimated for all subjects using a predictive equation integral to the Bod Pod® software. The calculated value for body density was used in the Siri equation [26] to estimate body composition. The complete body composition measurement was performed twice. If the %BF was within 0.05 %, the two tests were averaged. If the two tests were not within the 0.05 % agreement, a 3rd test was performed and, then, the average of three complete trials was used for all body composition variables.

### Exercise protocol

All participants followed a ramp exercise cycling protocol using an electromagnetically braked cycling ergometer (Schoberer Rad Messtechnik, SRM, Ergo, Julich, Germany) during their first visit. The test was initiated with a power output of 30 W in females and 50 W in males and was increased in a ramp fashion, with the cadence maintained at 65–70 rpm throughout the whole test, until reaching volitional exhaustion defined as 1) meeting the at least two of $\left(\overset{\cdotp }{\mathrm{V}},{\mathrm{O}}_{2\mathrm{peak}}\right)$ termination criteria: RER value > 1.1, heart rate within ten beats.min^−1^ of age-predicted maximum heart-rate, or achieving leveling-off of $\left(\overset{\cdotp }{\mathrm{V}},{\mathrm{O}}_2\right)$, or 2) the participant could no longer maintain the required cadence for over 15 s despite verbal encouragement. Similar verbal encouragement was provided to all participants throughout the exercise tests. The cycling ergometer was calibrated before use. The cycling positions that were taken in the first test were re-applied in the following visits.

The following two visits involved the participants to follow a 30 min exercise cycling test at their individually determined Fatmax intensity. Additionally, the heart rate (HR) was measured continuously (Polar Sporttester, Polar Electro, Finland) and the rate of perceived exertion (RPE) using the Borg scale (6–20) was measured every 3 min during all tests.

### Data processing and statistical analysis

The metabolic data of FAO and CHO were estimated using the stoichiometric indirect calorimetry equations (Eqs. 1 and 2), assuming minimal protein contribution during exercise.

1 $$ \mathrm{F}\mathrm{A}\mathrm{O} = 1.695 \times \kern0.5em \overset{\cdotp }{V}{O}_2\hbox{--} 1.701\kern0.5em \times \overset{\cdotp }{V}C{O}_2 $$

2 $$ \mathrm{C}\mathrm{H}\mathrm{O} = 4.585 \times \kern1em \overset{\cdotp }{V}C{O}_2\kern0.5em \hbox{--} \kern0.5em 3.226 \times \kern0.5em \overset{\cdotp }{V}{O}_2 $$

The FAO during exercise was determined based on averaging the last twenty min of the 30 min. Fatmax (g.min^−1^) was determined from the ramp test as the highest amount of FAO averaged over 30s. Fatmax corresponding intensity was determined as the power output (W) and relative intensity (%) relative to peak power (P_peak_), at which each participant achieved Fatmax [27].

Profile of mood state (POMS) questionnaire was analyzed for total mood disturbance total score, calculated for each participant by adding scores for Tension, Depression, Anger, Fatigue and Confusion and subtracting your Vigor score [28]. Hunger scale (1–10 scores) was used to indicate a satiety score, with a score of one being starving and ten being stuffed.

All data were described as means and standard deviations. FAO, CHO oxidation, and POMS questionnaire, were analyzed with 2x3 repeated measures ANOVA, to compare repeated conditions (SHRED vs. PL) over time (first hour pre, second hour pre, and post-exercise). The same procedure was used with Hunger Scale considering six time points (pre, every 30 min during exercise, and post), with Heart Rate and RPE considering 11 time points (rest, and every 3 min), and RPE considering ten time points (every 3 min). Post-hoc calculations were adjusted with Bonferroni method. Generalized Eta squared (*η*_G_^2^) and Cohen’s d were calculated as standardized effect size measures. Significance level was set at 0.05.

## Results

Fatmax determined from the baseline assessment was 0.89 ± 0.03 corresponding to a Fatmax intensity of 43.9 ± 8.6 % of P_peak_ (Table 1).

**Table 1:** Subject characteristics peak values

	Mean ± SD
HR_peak_ (BPM)	172.8 ± 13.1
P_peak_ (W)	174.7 ± 50.3
$\overset{\cdotp }{\mathrm{V}}{\mathrm{O}}_{2\mathrm{peak}}\mathrm{Z}$ (ml.kg^−1^.min^−1^)	32.4 ± 8.4
Fatmax (W)	76.8 ± 30.7
Fatmax (g.min^−1^)	0.89 ± 0.03
Fatmax (%)	43.9 ± 8.6

*HR*_*peak*_ Peak heart rate, *P*_*peak*_ Peak power output, $\overset{\cdotp }{\mathrm{V}}{\mathrm{O}}_{2\mathrm{peak}}$ Peak oxygen uptake, *Fatmax* Maximal fat oxidation, *%P*_*peak*_ Relative to peak power

### Fat oxidation

Significant effect on time was found [F(2,22) = 31.77, *p*< 0.001, *η*_G_^2^ = .54] but no significant effect on condition [F(1,11) = 2.31, *p* = 0.157, *η*_G_^2^ = .03, or interaction [F(2,22) = 3.18, *p* = 0.061, *η*_G_^2^ = .04] was revealed (Table 2).

**Table 2:** Results and ANOVA contrasts for FAO and CHO oxidation under each condition (*n* = 12)

	SHRED	PL	
	Mean ± SD	Mean ± SD	*p*
Fat oxidation (g.min^−1^)			
Pre 1	0.56 ± 0.26	0.67 ± 0.25	0.121
Pre 2	0.96 ± 0.37	0.74 ± 0.19	0.097
Post	3.80 ± 1.92	2.80 ± 2.02	0.120
CHO oxidation (g.min^−1^)			
Pre 1	2.73 ± 0.95	2.13 ± 0.70	0.064
Pre 2	2.48 ± 1.12	2.12 ± 0.80	0.274
Post	29.29 ± 11.19	30.54 ± 13.11	0.399

*M* Mean,*SD* Standard deviation, P Bonferroni-adjusted post-hoc contrast between SHRED and PL, *Pre1* 150 min before exercise, *Pre2* Immediately before exercise, *Post* Post exercise, *BPM* Beat per min

Post-hoc analysis determined significant increases in SHRED from Pre1 to Pre2 (*p* = 0.003 d = 1.34), Pre1 to Post (*p*< 0.001, d = 1.72) and Pre2 to Post (*p*< 0.001 d = 1.66), in PL from Pre1 to Post (*p* = 0.008, d = 1.12) and Pre2 to Post (*p* = 0.009, d = 1.09), but no difference from Pre1 to Pre2.

Although no significant *p*-values were found when comparing conditions (Pre1: *p* = 0.362, d = 0.27; Pre2: *p* = 0.096, d = 0.52; Post: *p* = 0.12, d = 0.49), Cohen’s d = 0.77 revealed moderate effect size in favor of SHRED from Pre to Post exercise.

### CHO oxidation

Significant effect on time was found [F(2,22) = 67.83, *p* < 0.001, *η*_G_^2^ = .79), but almost null effect on condition [F(1,11) = 0.025, *p* = 0.876, *η*_G_^2^ ≈ .00], and no significant effect on interaction [F(2,22) = 1.85, *p*= 0.181, *η*_G_^2^<.01) was revealed. Post-hoc analysis determined significant increase from Pre2 to Post (*p*< 0.001), but no difference from Pre1 to Pre2 (Table 2).

### RPE

Significant interaction [F(9.99) = 3.37, *p* = 0.001, *η*_G_^2^ < .01), time [F(9,99) = 27.81, *p* < 0.001, *η*_G_^2^ = .24), and condition [F(1,11) = 25.12, *p* < 0.001, *η*_G_^2^ = .06] effects were found. Post-hoc analysis revealed no significant difference between conditions at the start (*p* = 0.191). However, significant differences were found in the nine measures taken every 3 min during exercise (*p* values: 0.005, 0.01, 0.039, 0.013, 0.012, 0.001, 0.002; <0.001, <0.001).

### Mood state

No significant interaction [F(2,22) = 0.06, *p* = 0.944, *η*_G_^2^ <.01], time [F(2,22) = 0.65, *p* = 0.533, *η*_G_^2^ < .01] or condition [F(1,11) = 1.49, *p* = 0.247, *η*_G_^2^ = .02] effects were found.

### Hunger scale

There was no significant effect on interaction, [F(5,55) = 0.98, *p* = 0.437, *η*_G_^2^ < .01] or time [F(5,55) = 1.44, *p* = 0.225, *η*_G_^2^ = .04]. However, there was a trend, though not significant towards a condition effect for a higher satiety score in Shred compared with control, [F(1,11) = 3.58, *p* = 0.085, *η*_G_^2^ = .05]. Post-hoc analysis revealed significantly higher satiety score in SHRED compared with the control condition at 30 min (point 2), (*p* = 0.039). (Table 3)

**Table 3:** Results and ANOVA contrasts for total mood disturbance, hunger, heart rate, and exertion under each condition (*n* = 12)

	SHRED	PL	
	Mean ± SD	Mean ± SD	*p*
POMS (TMD + 100)			
Pre 1	97.7 ± 10.6	102.4 ± 13.1	0.170
Pre 2	99.2 ± 12.6	102.8 ± 17.2	0.493
Post	97.3 ± 11.4	100.5 ± 14.6	0.423
Hunger Scale (1–10 Score)			
Test start	4.3 ± 0.8	4.0 ± 0.6	0.191
30 min	4.25 ± 0.75	3.92 ± 0.67	0.039
60 min	4.33 ± 0.89	4.00 ± 0.60	0.166
90 min	4.17 ± 1.12	3.67 ± 0.49	0.166
120 min	4.33 ± 1.44	3.67 ± 0.78	0.171
Post	3.75 ± 1.29	3.50 ± 0.80	0.339
Heart Rate (BPM)			
Rest	67.5 ± 13.0	67.2 ± 9.7	0.903
3 min	106.5 ± 15.4	111.9 ± 12.8	0.090
6 min	112.4 ± 19.8	117.8 ± 17.0	0.110
9 min	118.9 ± 22.6	120.4 ± 20.9	0.620
12 min	121.6 ± 22.7	123.8 ± 20.5	0.473
15 min	124.4 ± 23.6	125.1 ± 21.1	0.816
18 min	124.6 ± 24.5	128.0 ± 21.6	0.348
21 min	128.2 ± 24.6	129.1 ± 20.1	0.814
24 min	129.4 ± 25.4	129.8 ± 21.3	0.895
27 min	132.3 ± 26.0	131.2 ± 22.1	0.758
30 min	131.6 ± 25.4	132.2 ± 22.0	0.865
RPE (scale 6–20)			
3 min	6.8 ± 1.4	7.0 ± 1.5	0.191
6 min	7.2 ± 1.5	8.1 ± 1.7	0.005
9 min	7.8 ± 1.8	8.7 ± 1.8	0.010
12 min	8.3 ± 1.9	8.9 ± 1.8	0.039
15 min	8.7 ± 2.1	9.3 ± 2.0	0.013
18 min	8.8 ± 2.0	9.6 ± 1.8	0.012
21 min	9.00 ± 2.0	10.1 ± 2.1	0.001
24 min	9.3 ± 1.9	10.4 ± 2.1	0.002
27 min	9.4 ± 1.9	10.6 ± 2.0	<0.001
30 min	9.4 ± 1.9	10.8 ± 2.0	<0.001

*M* mean, *SD* Standard deviation, *p* Bonferroni-adjusted post-hoc contrast between SHRED and PL, *Pre1* 150 min before exercise, *Pre2* Immediately before exercise, *Post* Post exercise, *BPM* Beat per min

### Heart rate

There was a significant effect on time, [F(10,100) = 70.97, *p*< 0.001, *η*_G_^2^ = .44]. However, no significant effect on condition [F(1,10) = 0.41, *p*= 0.536, *η*_G_^2^<.01] or interaction [F(10,100) =1.83, *p* = 0.065, *η*_G_^2^ <.01] was found.

## Discussion

The present study is the first to demonstrate an acute effectiveness of a multi-ingredient supplement, SHRED-Matrix for exercise-related metabolic outcomes. Findings include an enhanced FAO during exercise combined with an enhanced perceived exertion and improved side-effects associated with satiety, often reported with weight-loss supplements [9, 29]. Ingesting a dosage of 1.5 g of SHRED increased the reliance on FAO as a fuel source by 28 % (*p*< 0.05) immediately before exercise (Pre 2). A 26 % higher FAO (though not significant, *p* > 0.05) was found for SHRED than PL, and this was found to elicit a medium size effects (Table 2). These effects on substrate utilization were combined by a significant improvement in the rate of perceived exertion throughout the exercise duration, and a trend towards a decrease in the perception of hunger with SHRED and a significantly higher satiety scores at 30 min following the ingestion (Table 3). These combined effects of SHRED before and during exercise on metabolism, satiety and perceived exertion were independent of mood state, which was unchanged, and suggest a potential role for SHRED in enhancing exercise-related fat-loss outcomes.

There has been a surge in research into the potential weight loss effects for combining a number of ingredients in the present fat-loss product, in order to maximize their effectiveness. For example, Hoffman et al. [10] found higher energy expenditure at rest when testing a weight loss supplement in healthy adults of a similar young-adult age to those in the present study, and containing similar ingredients such as anhydrous caffeine, Yerba Maté extract and yohimbine. The increased reliance on FAO at rest in SHRED compared with PL found in the present study support the thermogenic role of these multi-ingredients in both products. However, the increased FAO as indicated by the moderate effect size, during moderate to heavy intensity exercise (Fatmax intensity of 44 % of P_peak_), (Table 1) suggests that the metabolic weight loss outcomes at rest can be further augmented during exercise.

The participants exercised at an individually determined Fatmax intensities, estimated from the first visit at an average of 44 ± 9 % (Table 1) relative to peak power, which is in line with what was previously reported for Fatmax intensities in healthy adults [27, 30]. Exercising at this intensity reflects an increased reliance on FAO as a fuel source and less reliance on CHO [15, 18]. Although, the condition (SHRED vs. PL) effects were not significant based on the ANOVA main effects, there was a medium effect size for the condition in favor of SHRED from Pre to Post exercise. The difference was 28 % for Pre and 26 % for Post indicating a possible augmented FAO when exercising at the Fatmax-intensity (Table 2). This difference is close to 24 % increase in FAO reported following Yerba Maté ingestion, one of the key ingredients in SHRED, and found over a similar exercise intensity range of 40–70 % of $\overset{\cdotp }{\mathrm{V}}{\mathrm{O}}_{2\mathrm{peak}}$ [23]. The trend in an enhanced FAO was supported by a reduced perception of exertion (Table 3). To our knowledge, this is the first study to demonstrate a potentially augmented FAO at constant load exercise (30 min) corresponding to Fatmax, following the ingestion of such a multi-ingredient product, which implies a favorable exercise-related fat loss, due to a further increase in the reliance on FAO beyond that when exercising alone.

It is likely that the multi-ingredients contained within the presently tested SHRED ingredient (Green Tea Extract, Yerba Maté, Guarana Seed Extract, Anhydrous caffeine, Saw palmetto, Fo-Ti, Eleuthero root, Cayenne Pepper, and Yohimbine) have had joint metabolic effectiveness in increasing the reliance on FAO, which can be further augmented during exercise found in the present study. Caffeine is perhaps the most studied single supplement for its effects on exercise performance for almost four decades [31]. Combining caffeine with other ingredients such as tea catechins in green tea, which promote energy expenditure [32] and caffeoyl derivatives (e.g. chlorogenic acid, caffeic acid) phytosterols and saponins in Yerba Maté, which promote fat metabolism [23], Capsaicinoids in Cayenne pepper, which promote satiety [33] and the natural alpha-2 antagonist, yohimbine which is considered to promote sympathetic activity by central and peripheral mechanisms to promote fat oxidation [22, 34]. Combining a mixture of previously known effective active ingredients may explain a number of resulting metabolic, neuromuscular, and hormonal changes that have resulted in increased energy expenditure, fat metabolism, and exercise performance enhancement [5, 9, 10]. RMR and EE has been reported to increase significantly at 60–180 min following ingesting products containing Yohimbine, green tea and caffeine, in the form of multi-capsules [22] and within an energy drink [35]. This resting duration is similar to the 150 min resting period used in the present study. It has been reported that green tea extract rich in catechin polyphenols and caffeine increases 24-h energy expenditure and fat oxidation at rest [32]. Similar thermogenic effects at rest have been reported from ingesting Green tea [32, 35-37], Caffeine [32, 38], and Citrus aurantium [39], all contained within the SHRED supplement that have been used in the present study.

It is reasonable to suggest that active ingredients combined in SHRED can be responsible for alleviating hunger, enhancing satiety and possibly psychomotor-related mood state response [8, 9, 11, 12]. Cayenne pepper, Yerba Maté, green tea, guarana, all can reduce appetite and energy intake, and improve mood state [24, 40, 41]. For example, Campos et al. [41] reported a reduce depression and improved physical performance in mice. Outlaw et al. [11] reported no main effects in POMS scores of alertness, focus energy and fatigue, with no changes in hunger scores following 1 h of ingesting a thermogenic mix containing some similar ingredients to SHRED such as caffeine, Yerba Maté, green tea, guarana, and black pepper. However, they reported a significant increase in both resting metabolic rate and energy expenditure. This is in line with a the trend towards an increased FAO without affecting the overall POMS score of total mood disturbance in both resting and exercise conditions found in this study (Table 3). Reinbach et al. [40] reported a suppressed hunger and increased satiety by ingesting a combination containing capsaicin and green tea, but their supplementation regime was over a three weeks period and the improved satiety was only found when a positive energy balance was followed by their participants. In comparison to our appetite results from the hunger scale, there was a trend towards an overall effect (*p* = 0.085), which was significant at 30 min (*p* < 0.039). The acute effects found on appetite in the present study are likely to have resulted from the active ingredients within the supplement we tested. Only limited data are available to confirm these effects, with one study reporting Yerba Maté effectiveness on appetite and satiety markers glucagon-like peptide 1 (GLP-1) and leptin in high-fat diet-fed mice [24]. Long term studies are needed to establish the appetite reduction effect of multi-ingredient weight loss products.

It may not be possible to compare the bioavailability of the several active ingredients within this multi-ingredient product during exercise. However, it is reasonable to actuate the metabolic outcomes based on understanding of the well-established concepts in the area of exercise metabolism [15, 18, 27]. Comparing the various effects of different commercially available multi-ingredient products during exercise may not be possible, even though their effectiveness has been comprehensively reviewed for weight loss [29]. It remains to be investigated how single ingredients and their concentrations within the multi-ingredients can be effective for augmenting weight-loss outcomes during exercise. The study focused on the combined effects of the multi-ingredients, but the individual ingredients were not studied here. While it is likely that they promoted some of the beneficial effects found, this study did not demonstrate whether they have worked synergistically. Future research may investigate the synergy of the multi-ingredients within such a fat-loss product.

## Conclusion

The present study demonstrated a potential acute effectiveness for ingesting SHRED on fat-loss outcomes after at least 30-min rest and during exercise at workloads corresponding to an individualized Fatmax-intensity. There is a trend of augmented FAO in SHRED compared with PL as indicated by moderate effect size in FAO. Fatmax exercise intensities are known markers for effective aerobic exercise prescription. Therefore, the multi-ingredient effects of SHRED on FAO, combined with a decreased perception of effort and a trend towards an improved satiety, suggest a potential acute effectiveness in improving exercise-related fat loss outcomes. Future research may investigate the chronic effects of multi-ingredient fat-loss products when combined with exercise on weight loss outcomes.

## Abbreviations

FAO: Fatty acid oxidation
CHO: Carbohydrates
RER: Respiratory exchange ratio
EE: Energy expenditure
RMR: Resting metabolic rate
COP: Cross-over point
Fatmax: Maximal fat oxidation
$$ \overset{\cdotp }{\mathrm{V}}{\mathrm{O}}_2\mathrm{Z} $$: Oxygen uptake
$\overset{\cdotp }{\mathrm{V}}\mathrm{C}{\mathrm{O}}_2\mathrm{Z}$: Carbon dioxide production
PER: Respiratory exchange ratio
P_peak_: Peak Power
$$ \overset{\cdotp }{\mathrm{V}}{\mathrm{O}}_{2\mathrm{peak}}\mathrm{Z} $$: Peak $$ \overset{\cdotp }{\mathrm{V}}{\mathrm{O}}_2 $$
HR_peak_: Peak heart rate
P_peak_: Peak power output
%P_peak_: Relative to peak power
BPM: Beat per minute
